# The protective effect of sulforaphane on type II diabetes induced by high‐fat diet and low‐dosage streptozotocin

**DOI:** 10.1002/fsn3.2040

**Published:** 2020-12-10

**Authors:** Shuhua Tian, Xiangfei Li, Yunfan Wang, Yingjian Lu

**Affiliations:** ^1^ College of Food Science and Engineering Nanjing University of Finance and Economics Nanjing China

**Keywords:** glucose intolerance, intestinal flora, NAFLD, sulforaphane, type II diabetes

## Abstract

Sulforaphane (SFN) which is abundant in broccoli florets, seeds, and sprouts has been reported to have beneficial effects on attenuating metabolic diseases, such as antiobesity, antidiabetes, and antioxidative activities. However, the effects of SFN on the regulation of type II diabetes through easing nonalcoholic fatty liver (NAFLD) and repairing pancreas tissue are rarely reported. In this study, we found that the administration with different dosages of SFN was able to increase serum insulin level, enhance HOMA‐β index, decrease fasting blood glucose and serum total cholesterol, triglyceride, low‐density lipoprotein (LDL‐C), fibroblast growth factor21 (FGF21) levels, ease NAFLD level, and repair the pancreas tissue. In addition, SFN was able to increase liver antioxidant capacities. In particular, high (10 mg/kg) dosage of SFN exerted a significant beneficial effect for decreasing serum lipopolysaccharide levels. Furthermore, the administration of SFN could also decrease the relative abundance of *Allobaculum* at the genus level. Low dosage (2 mg/kg) of SFN could increase the relative abundance of *Bacteroidetes* and decrease the relative abundance of *Firmicutes* at the phylum level. Overall, our results showed that SFN exerted its antidiabetic effect through easing NAFLD and repairing pancreas tissue in association with modulation of gut microbiota. The ease of NAFLD by SFN was accompanied by enhancing liver antioxidant abilities and improving FGF21 resistance.

## Graphical abstract

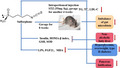

## 1 INTRODUCTION

There are currently more than 150 million people with diabetes worldwide, and there will be 366 million people with diabetes around the world at 2030s (Shaw et al., 2010). The majority of people with diabetes in China are type II diabetes, usually between the ages of 35 and 40 (Saeedi et al., 2019). Type II diabetes mainly caused by insulin resistance or minor pancreas damage is characterized by hyperglycemia. In the development of insulin resistance, blood glucose levels do not rise significantly until islet β‐cell is damaged and insulin secretion begins to decrease (Tushuizen et al., 2007). It is well known that long‐term high‐fat diet can cause insulin resistance which will develop into liver and pancreas fatification (Heiskanen et al., 2018). Fatty pancreas can lead to damage to islet cells which in turn leads to a decrease in insulin secretion (Lim et al., 2011). In addition, fatty liver can lead to the decrease of glucose metabolism in the liver tissue which in turn exacerbates the incidence of diabetes (Ryan et al., 2013). Recent evidences have shown that bioactive compounds extracted from natural plants can protect mice from type II diabetes. For instance, it has been reported that cinnamon polyphenols, edgeworthia gardneri flos, and shubat protect diet‐induced diabetes through repairing impaired pancreas (Chen & Zhan, 2019; Liao et al., 2019; Manaer et al., 2015). Furthermore, the mixed lactobacillus and sea cucumber oligopeptide cannot only effectively alleviate hyperglycemia but also alleviate the liver antioxidant ability of diabetes (Li et al., 2018; Wang et al., 2019).

The intestine is the main area of human material metabolism and nutritional absorption, the total amount of intestinal bacteria is almost 10 times that of human cells, forming a total weight of about 1.5 kg of the flora intestinal microecosystem (Xu & Gordon, 2003). There are a wide variety of intestinal microorganisms; the largest flora is *Firmicutes* and *Bacteroides* (Eckburg et al., 2005). As the second genome acquired by human beings, the intestinal flora interacts with the human body, affecting the physiological functions of the body, such as nutrition, metabolism, and immunity. Previous studies have demonstrated that the imbalances of intestinal flora may increase the rate of obesity, genetics, islet dysfunction, insulin resistance, and type II diabetes (Ma et al., 2019). Therefore, it is important to understand the relationship between gut microbes and hosts, and to provide effective strategies for human beings to prevent diabetes and other metabolic diseases.

Sulforaphane derived from the hydrolysis of glucoraphanin has been reported to have important medicinal value. SFN has been reported to exhibit antioxidant, neuroprotective and anticancer properties (Guerrero‐Beltran et al., 2012; Liang & Yuan, 2012). It has also been reported that SFN has a potential effect to fight obesity by activating AMPK signaling pathway (Choi et al., 2014; Lee et al., 2012; Yao et al., 2015). SFN has been used for a long time in reducing hyperglycemia activity. In the previous study, SFN was reported to improve insulin sensitivity but did not change the antioxidant response of diabetic rats (Souza et al., 2016). In addition, Axelsson et al. found that SFN can reduce the production of glucose in hepatocytes induced by palmitate in (Axelsson et al., 2017). SFN was also reported to protect diabetic retinopathy through activating of the Nrf2 pathway and inhibiting of NLRP3 inflammasome formation (Li et al., 2019). Moreover, despite of the improving hyperglycemia, the mechanism involved in easing NAFLD and repairing pancreas tissue on ameliorating type II diabetes are not observed, and direct evidence for the effect of the SFN on regulating the gut microbiota is rarely reported. Thus, the aim of this study was to estimate the antidiabetic effect of SFN and to explore whether SFN could protect high‐fat and STZ‐induced type II diabetes by easing NAFLD and repairing pancreas tissue. Additionally, the 16S rRNA‐V4 gene sequencing technique is used to understand whether SFN could show the improvement of intestinal flora which is involved in type II diabetes.

## 2 CHEMICALS AND METHODS

### 2.1 Chemicals and reagents

Sulforaphane (Sigma Chemical Co.), Dimethyl sulfoxide (Aladdin), Insulin (Yuanye Biotechnology), and PBS phosphate buffer (Solable) were obtained from different chemicals companies in the United States and China.

### 2.2 Animal experiments and methods

The high‐fat diet (D12492) was purchased from the American Research diet company. Animal experimental environment (temperature 20°C–23°C, humidity 40%–70% [(50 ± 5)%], wind speed 0.1 m/s–0.2 m/s, airflow ≥ 28 times/min) were provided by Nanjing Agricultural University (SYXK 2017‐0007). After 2 weeks of acclimation, 60 mice were separated into six groups: CN: mice fed low‐fat diet; DM: mice fed high‐fat diet; PC: mice fed high‐fat diet plus 300 mg/kg metformin; LS: mice fed a high‐fat diet plus 2 mg/kg SFN; HS: mice fed high‐fat diet plus 10 mg/kg SFN; DS: mice fed high‐fat diet plus 1.875% DMSO. SFN was dissolved in 1.875% DMSO. After eight weeks of gavage, mice were fasted for 12 hr. Then, DM, PC, LS, and HS groups injected with STZ (55 mg/kg) (Sigma Chemical Co.) which was dissolved in 1x PBS buffer, while CN group injected with an equivalent volume of PBS buffer. After intraperitoneal injection, mice were gavaged for another four weeks. At week 10, the fasting blood glucose (FBG) level in the DM group was higher than 11.1 mM which was confirmed as a type II diabetes model (Gao et al., 2015). Mice feces are collected at the last week. At the end of 12 weeks, the mice were fasted for 12 hr to execute by dislocation. Blood samples were taken from eyeball and prepared for serum analysis by centrifugation at 850 *g* for 15 min. The livers, pancreas, and epididymal white adipose (eWAT) were collected, weighted, and freezed at −80°C.

### 2.3 Serum biochemical analysis

Low‐density lipoprotein (LDL‐C), high‐density lipoprotein (HDL), total cholesterol (TC), triglyceride (TG), malondialdehyde (MDA), superoxide dismutase (SOD), glutathione (GSH), and glutathione peroxidase (GSH‐Px) were measured according to the commercial kits (Nanjing Jiancheng Biology Engineering Institute). Insulin, lipopolysaccharide (LPS), and fibroblast growth factor21 (FGF21) were measured according to the commercial kits (Elabscience Biotechnology, WuHan, Hubei, China). Homeostasis model assessment‐β (HOMA‐β) = (20 × FINS)/(FBG − 3.5) (Haffner et al., 2002).

### 2.4 Histological analysis

Specimens of liver, eWAT, and pancreas tissues were collected and fixed in 10% buffered formalin for 48 hr. After the successful tissue fixation, it needs to be pruned to 25px * 25px * 5px, placed in a packed box, and rinsed with flowing water (remove formalin from the tissue) for 30 mins. The tissue blocks were placed in the transparent agent xylene which was able to dissolve alcohol and paraffin wax. Then, the transparent tissue was put into the melt paraffin and insulated in the wax box, encapsulated after the paraffin completely immersed into the tissue blocks. Finally, the blocks were sectioned at 5 µm, deparaffinated and stained with hematoxylin and captured using an Olympus CX41 camera (Olympus).

### 2.5 Analysis of intestinal flora

Four mice were selected from every group casually for intestinal flora analysis. Weighed cecum (100 mg) from each mouse were pretreated and tested by BGI (The Beijing Genomics Institute) for amplification on the V4 region of 16sRNA. The analysis was based on species annotation and OTU results through high‐throughput sequencing.

### 2.6 Statistical analysis

The results were presented as means ± *SEM* (*n* = 8). SPSS 24.0 was used in the expression of experimental results. All groups were analyzed using a one‐way analysis of variance (ANOVA) with Tukey's HSD post hoc test (*p < *.05). Statistical significance was declared at *p* < .05.

## 3 RESULTS AND DISCUSSION

### 3.1 Effect of Different Dosages of SFN on body weight, organ coefficient in type II diabetes

**Figure 1:**
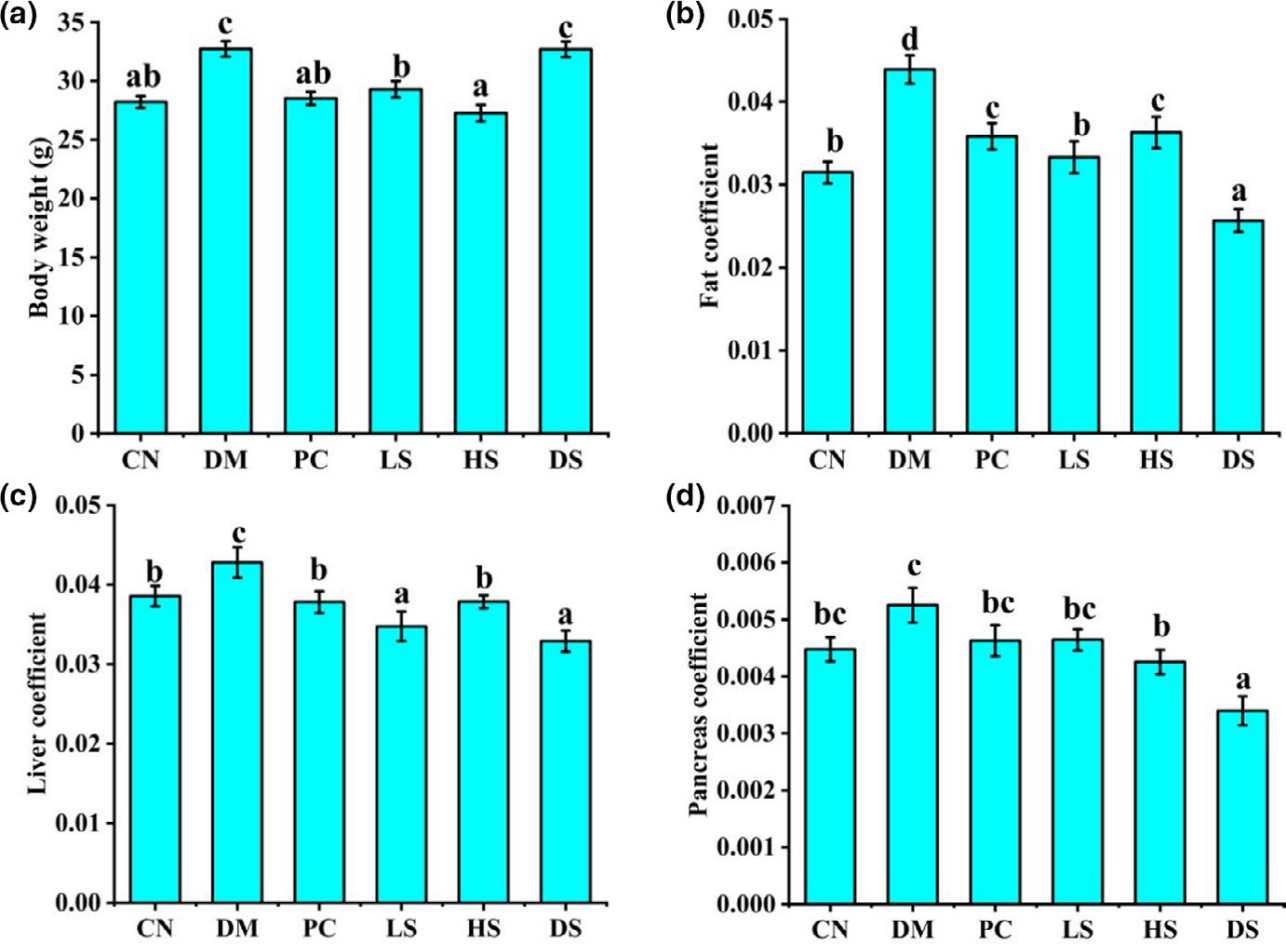
Effect of different dosages of SFN on body weight, organ coefficient in type II diabetes. (a) Body weight. (b) Fat coefficient. (c) Liver coefficient. (d) Pancreas coefficient. Value is the mean ± *SEM* (*n* = 8). Data not sharing a common superscript differ significantly among groups (*p* < .05, ANOVA)

Most of patients with type II diabetes have a “history” of being overweight and their weight is lower during the development of disease, but it is less pronounced due to their large weight base (Beebe, 2003). Our result demonstrated that in comparison with the CN group, the body weight of diabetic mice was markedly higher than that of the CN group. Oral supplementation of SFN and metformin had a significant lower body weight compared to DM group (Figure 1a). In addition, diabetes mice were found to significantly raise the levels of fat and liver coefficients. Especially, oral administration of different dosages of SFN and metformin all remarkably recovered the levels of fat coefficient and liver coefficient compared to the DM group (Figure 1b,c). However, high dosages of SFN remarkably reduced the pancreas coefficient level compared to CN group (Figure 1d). Both of our body weight and organ coefficient results demonstrated that SFN and metformin had the beneficial effects on fighting with overweight which was consistent with previous studies.

### 3.2 The effect of different dosages of SFN on serum lipid in type II diabetes

Serum lipid metabolism disorder serotonin is a key reason for type II diabetes and is also the main cause of cardiovascular disease (Chen et al., 2014). Shubat was demonstrated to decrease serum TC, TG, and LDL‐C levels and increase HDL‐C level in type II diabetes (Manaer et al., 2015). Previous study has demonstrated that SFN was able to decrease serum TC level; however, it had no significant influence on serum TG, LDL‐C, and HDL‐C levels compared to diabetic mice (Choi et al., 2014). As presented in Table 1, the DM group contained markedly higher concentrations of TC, TG, and LDL‐C levels compared to the CN group. The treatment of LS and HS led to a significant reduction in the levels of TC, TG, and LDL‐C. However, the level of HDL‐C in the HS group was markedly higher compared to DM group. Thus, SFN showed the significant effects on improving the serum lipid index in type II diabetes.

**Table 1:** Effect of different dosages of SFN on TC, TG, HDL‐C, and LDL‐C levels

Items	TC (mM)	TG (mM)	HDL‐C (mM)	LDL‐C (mM)
CN	9.98 ± 0.54^a^	1.01 ± 0.12^a^	8.22 ± 0.40^a^	0.56 ± 0.07^a^
DM	14.15 ± 1.09^c^	1.45 ± 0.13^b^	9.33 ± 0.19^b^	1.58 ± 0.11^d^
PC	13.11 ± 1.64^bc^	1.02 ± 0.04^a^	7.90 ± 0.44^a^	0.80 ± 0.13^b^
LS	12.55 ± 0.94^b^	0.99 ± 0.09^a^	8.18 ± 0.32^a^	0.85 ± 0.06^b^
HS	10.01 ± 0.50^a^	1.01 ± 0.06^a^	10.25 ± 0.88^b^	0.79 ± 0.06^b^
DS	11.09 ± 0.79^a^	1.32 ± 0.13^b^	11.23 ± 0.55^c^	1.22 ± 0.12^c^

Note

Value is the mean ± *SEM* (*n* = 8). Data not sharing a common superscript differ significantly among groups (*p* < .05, ANOVA).

Abbreviations: HDL, high‐density lipoprotein cholesterol; LDL‐C, Low‐density lipoprotein cholesterol; SFN, sulforaphane; TC, total cholesterol; TG, triglyceride.

### 3.3 Effects of different dosages of SFN on glucose tolerance and FBG in type II diabetes

**Figure 2:**
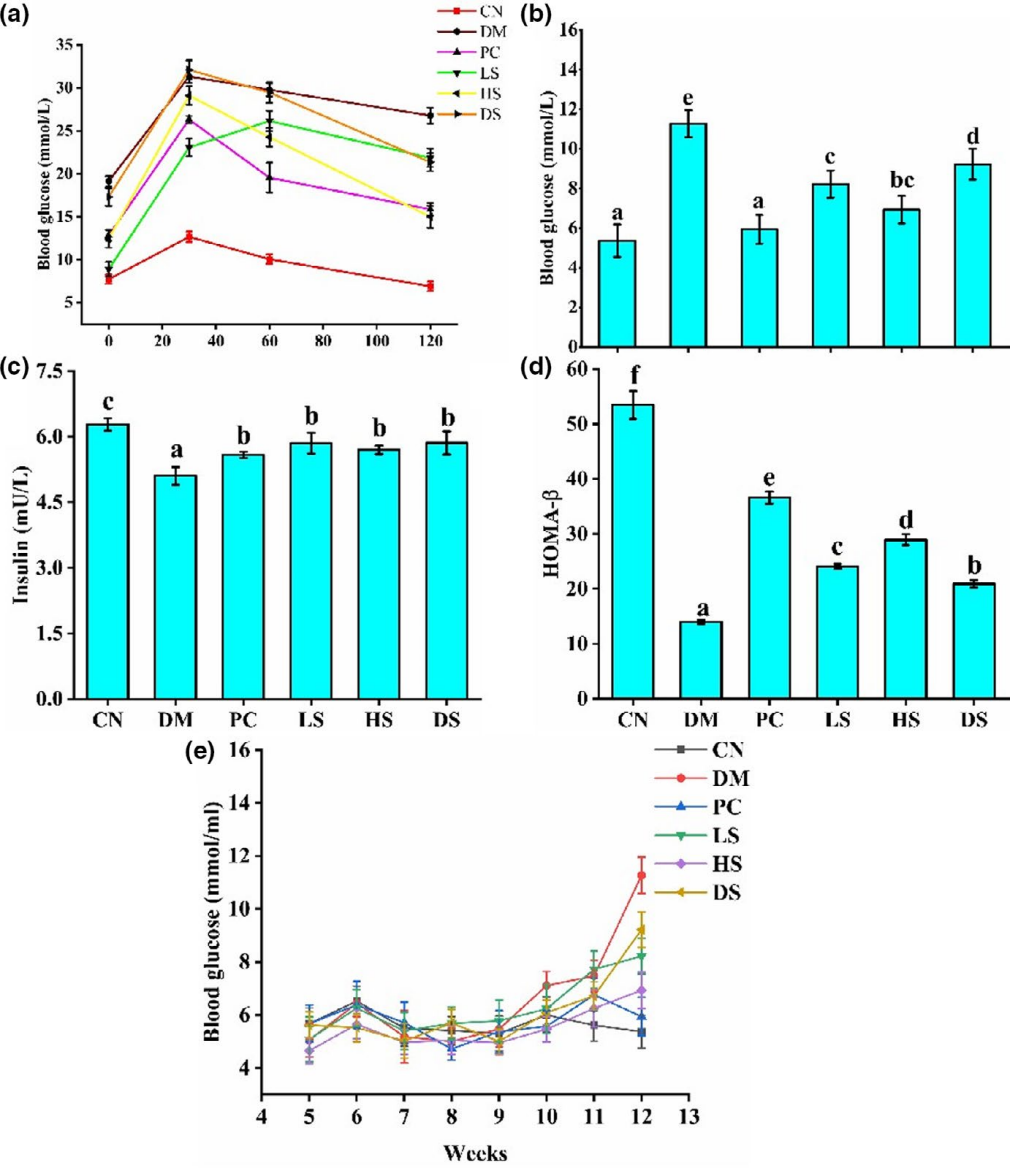
Effect of different dosages of SFN on (a) Oral glucose tolerance. (b) Fasting serum glucose levels. (c) Fasting serum insulin levels. (d) HOMA‐β. Value is the mean ± *SEM* (*n* = 8). Data not sharing a common superscript differ significantly among groups (*p* < .05, ANOVA).

Currently, the oral glucose tolerance (OGTT) test is used to determine β‐cell function in clinical practice. It has been reported that SFN can reduce hepatic glucose production and improves glucose tolerance in patients with type II diabetes (Axelsson et al., 2017). At week 12, OGTT (2 mg/kg glucose by gavage) was performed in overnight‐fasted mice for every mouse. The results of blood glucose levels at 0, 30, 60, and 120 min were depicted in Figure 2a. The blood glucose levels of the DM group were higher than that of the CN group at 0, 30, 60, and 120 min, respectively **(**Figure 2a). Oral administration of HS and PC revealed a decrease at the points of 60 and 120 min.

Fasting blood glucose level of the DM group was significantly higher than that of the CN group. Supplement of PC, LS, and HS to the diabetic mice generated a positive effect on hypoglycemic action. However, the FBG levels of the mice in the DM and DS groups showed no significant differences (Figure 2b). The fasting blood insulin level of the DM group was lower than CN, PC, and HS groups. The PC and HS administration significantly increased the fasting blood insulin levels (Figure 2c). HOMA‐β is an indicator used to evaluate the function of islet β‐cell in individuals. For patients with diabetes, the HOMA‐β index deviates from normal values due to different disease progressions. The function of islet β‐cell decreases when diabetes becomes seriously (Daems et al., 2019). As the result showed, the HOMA‐β index in the DM group was pronounced reduced compared to the CN group. However, PC, LS, and HS administration could markedly increase HOMA‐β index (Figure 2d). Based on the results of fasting blood insulin level and HOMA‐β index, the islet β‐cell function was impaired in the DM group, and the supplement of PC, LS, and HS to the diabetic mice could attenuate glucose intolerance and decrease FBG by repairing islet β‐cell function. Figure 2e showed the changes in the FBG level of all groups. After 1 week of STZ injection, the FBG levels of the PC, LS, DM, and HS groups did not significantly increase, but from week 10, the blood glucose levels of the PC, LS, and HS groups showed a significant increase compared to CN group, the blood glucose of the DM group was more than 11.1 mM, which met the standard for type II diabetes (Gao et al., 2015). However, the blood glucose of LS, HS, and PC groups was lower than DM group, it contributed to the protective effect of SFN on type II diabetes.

### 3.4 Effects of different dosages of SFN on liver antioxidant capacity in type II diabetes

**Figure 3:**
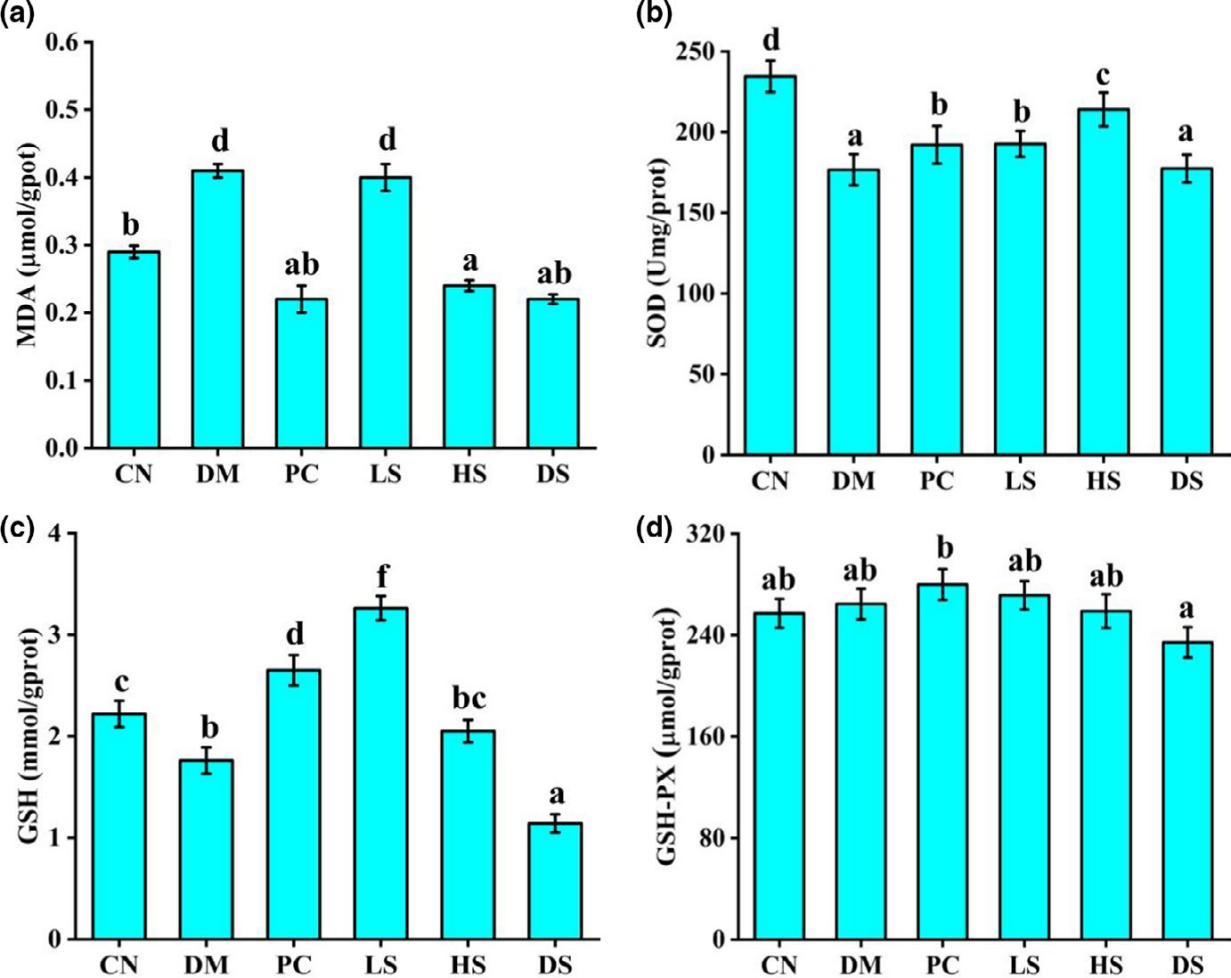
Effects of different dosages of SFN on liver antioxidant capacity in type II diabetes. (a) Effect of SFN on liver MDA. (b) Effect of SFN on liver GSH. (c) Effect of SFN on liver SOD. (d) Effect of SFN on liver GSH‐PX. Value is the mean ± *SEM* (*n* = 8). Data not sharing a common superscript differ significantly among groups (*p* < .05, ANOVA)

The development of type II diabetes is accompanied by oxidative stress and the destruction of the antioxygenation system (Betteridge, 2000). The SFN protect mice from oxidative stress may through activating Nrf2/Keap1 pathway (Calabrese et al., 2012). In addition, SFN has been reported to protect cells against high glucose‐induced oxidative stress by decreasing the TNF‐*α* and IL‐6 levels, and increasing GSH, SOD, and CAT activities (Li et al., 2019). The MDA in liver of the CN group was significantly lower than DM group (Figure 3a). However, GSH and SOD activities in liver of the CN group were higher than DM group (Figure 3b,c). Following treatment with LS and HS, the activity of MDA decreased significantly. In particular, high dosage of SFN was better at decreasing liver MDA. When treated with PC, LS, and HS, the activity of GSH and SOD was significantly increased compared to DM group. However, there was no significant difference between CN, DM, PC, LS, HS, and DS groups in liver GSH‐PX activity (Figure 3d). Hence, the administration of PC, LS, and HS was effective for the improvement of liver antioxidant ability.

### 3.5 Effects of different dosages of SFN on serum FGF21 and LPS level in type II diabetes

**Figure 4:**
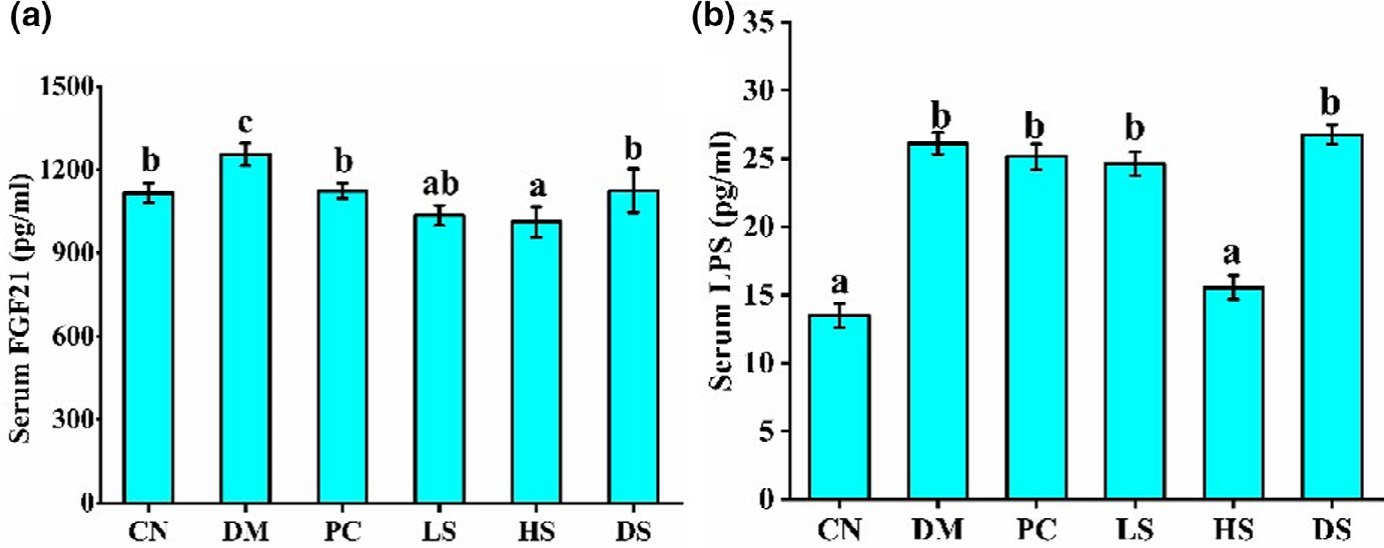
Effects of different dosages of SFN on serum FGF21 and LPS level in type II diabetes. (a) Serum FGF21 level. (b) Serum LPS level. Value is the mean ± *SEM* (*n* = 8). Data not sharing a common superscript differ significantly among groups (*p* < .05, ANOVA)

Numerous studies have shown that FGF21 has a significant beneficial effect on regulating glucose and lipid metabolism, including increased insulin sensitivity, reduced triglyceride levels, attenuating hepatic steatosis and reduced blood glucose (Kharitonenkov et al., 2005). Previous studies have reported that white pitaya (hylocereus undatus) juice produced the antidiabetic and anti‐NAFLD effect by improving FGF21 resistance (Song et al., 2015). Our result demonstrated that DM group had a significantly higher concentration of serum FGF21 level than other groups; PC, LS, and HS treatment could markedly decrease serum FGF21 level (Figure 4a). Therefore, SFN could alleviate FGF21 resistance, which had beneficial effects on improving NAFLD and diabetic symptoms. LPS is an endotoxin that affects insulin signaling pathways through the JNK pathway by activating inflammation reflection (Cani, Amar, et al., 2007). In addition, the accumulation of serum LPS resulted in body weight gain and eventually increased the risk of type II diabetes (Cani, Neyrinck, et al., 2007). Serum LPS level of all diabetic mice was significantly higher than those of the CN mice, supplement with PC, LS, and DS had no beneficial effect on reducing serum LPS concentration. However, HS supplement had a marked effect on reducing serum LPS concentration which had beneficial effect on easing type II diabetes (Figure 4b).

### 3.6 Effect of SFN on NAFLD in type II diabetes

**Figure 5:**
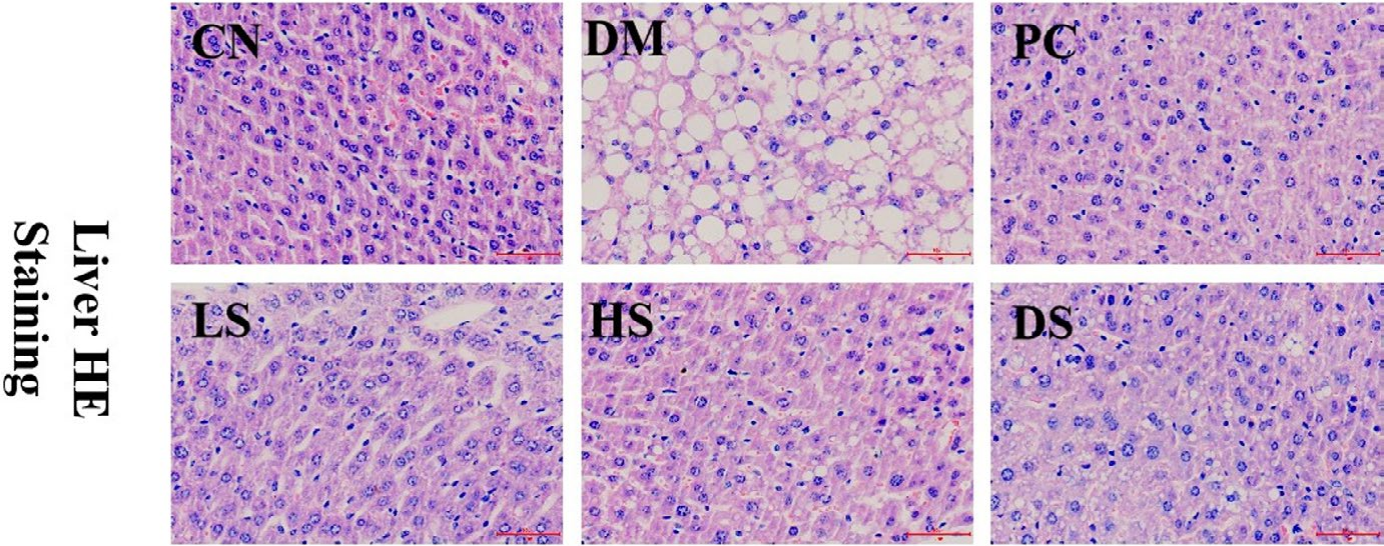
Effect of SFN on fatty liver in type II diabetes. Value is the mean ± *SEM* (*n* = 4)

It has been reported that liver lipid accumulation is a marker of liver steatosis, which is associated with oxidative stress and inflammation (Charlton, 2004). Liver impairment and steatosis will influence glucose metabolism and aggravate type II diabetes. SFN was reported to attenuate HFD‐induced visceral adiposity, adipocyte hypertrophy, and liver fat accumulation (Choi et al., 2014). Our study was conducted to assess the effect of SFN supplement on type II diabetes‐related NAFLD. H&E staining of livers revealed an obvious hepatic lipid droplet accumulation in diabetic mice. When treated with LS, HS, and PC, the hepatic lipid droplets were pronounced reduced, which indicated that LS, HS, and PC administration had the potential to alleviate NAFLD (Figure 5). Based on our result of liver H&E staining with liver antioxidant capacity and serum FGF21 level, we speculate that SFN may restore its glucose metabolism function by easing NAFLD which involved in enhancing of liver antioxidant ability and attenuating FGF21 resistance.

### 3.7 Effect of different dosages of SFN on the pancreas dysfunction in type II diabetes

**Figure 6:**
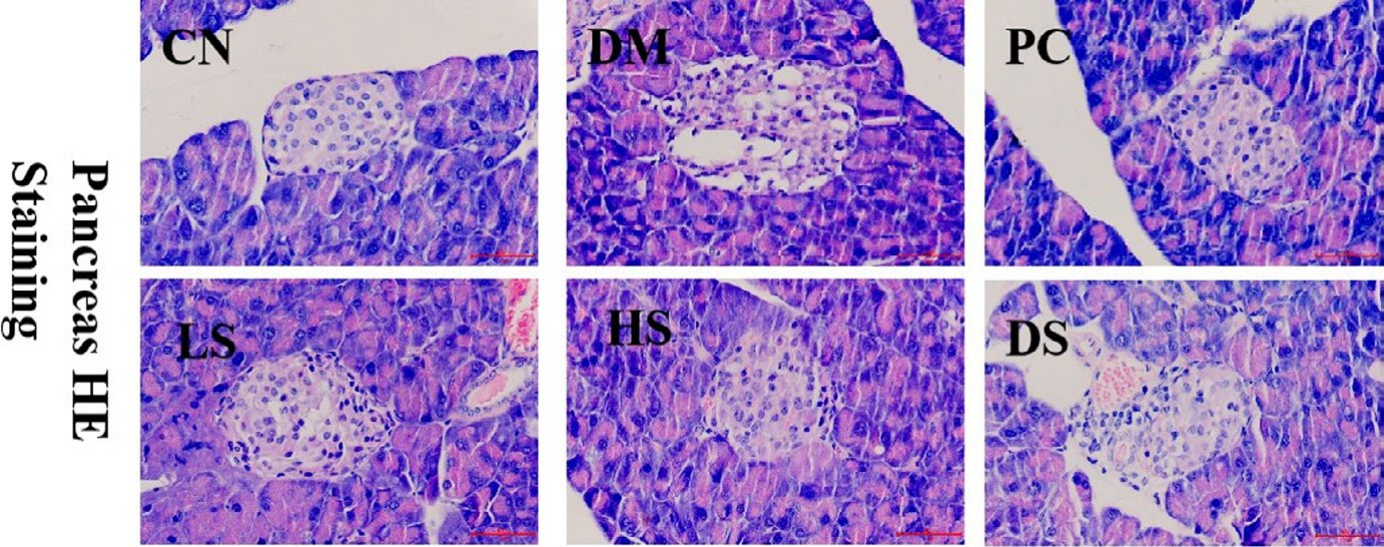
Effect of different dosages of SFN on the pancreas in type II diabetes. Value is the mean ± *SEM* (*n* = 4)

A long‐term high‐fat diet caused the augmentation of body adipocyte tissue, such as pancreas and liver lipid droplets accumulation (Heiskanen et al., 2018). In addition, a small dosage of STZ (55 mg/kg) induces mild apoptosis of pancreatic cells. Both high‐fat diet and a small dosage of STZ (55 mg/kg) contributed to islet β cells dysfunction. H&E staining of the pancreas suggested that the lipid droplets were very serious in islet cells and the number of pancreatic cells was impaired in the DM group compared to that in the CN group. DM mice treated with LS, HS, and PC showed an improvement of severe lipid bubbles to a certain extent (Figure 6). After the destruction of pancreatic β cells, the function of β cells will be impaired and the HOMA‐β index will be decreased (Daems et al., 2019). Our results demonstrated that the easing of lipid droplets accumulation in pancreas tissue by SFN led to higher glucose tolerance, HOMA‐β index, and insulin secretion.

### 3.8 Effect of different dosages of SFN on intestinal flora in type II diabetes

**Figure 7:**
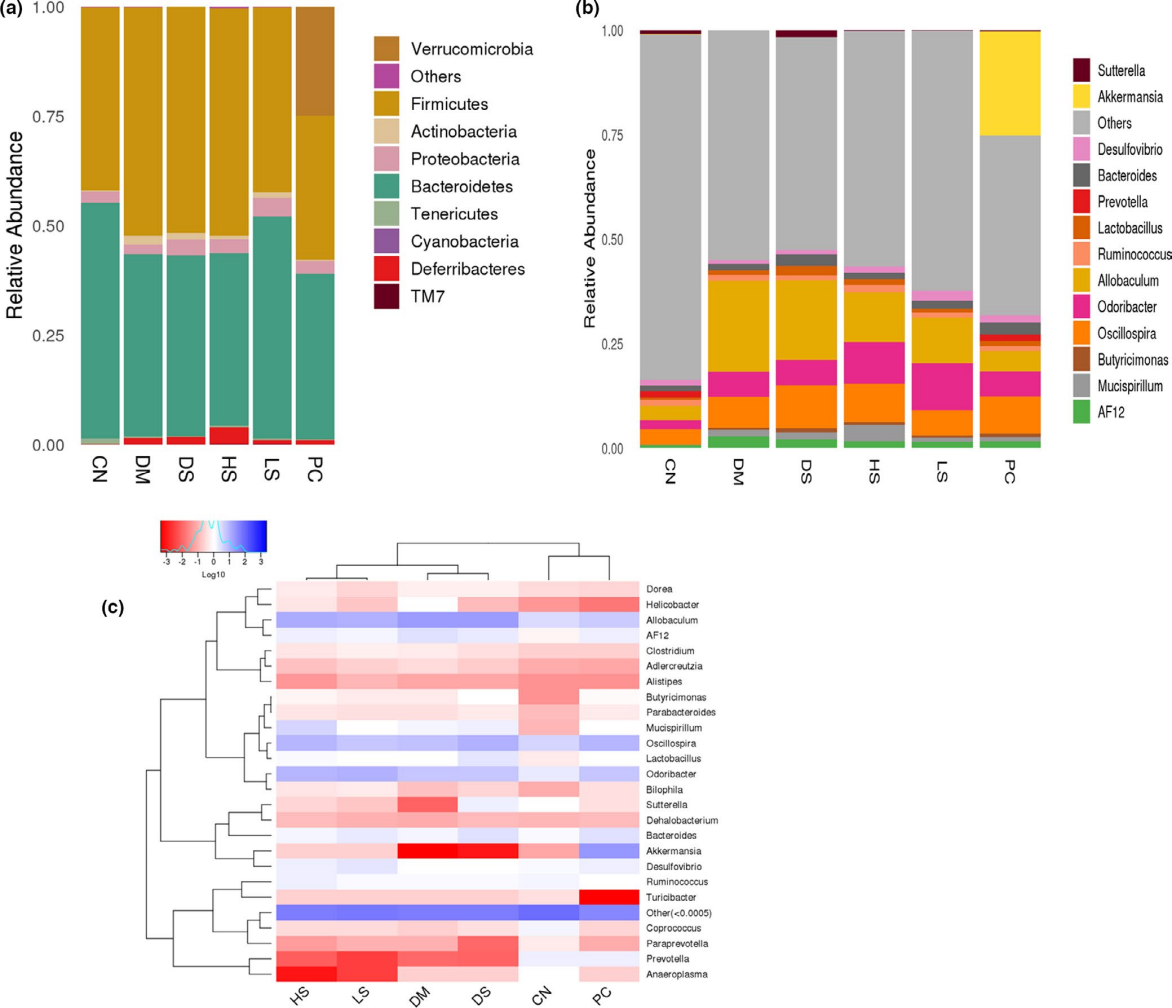
Effect of different dosages of SFN on intestinal flora in type II diabetes. (a) The composition and relative abundance of bacterial communities at the phylum level. (b) Heatmap analysis of the microbiota changes with different dietary interventions at the genus level. Value is the mean ± *SEM* (*n* = 4)

Intestinal flora is significant in regulating the energy metabolism of host cells and the changes of intestinal flora induce various metabolic diseases, such as obesity and type II diabetes (Qin et al., 2012; Turnbaugh et al., 2006). Numerous studies have reported that the number of *Bifidobacteria*, *Clostridium,* and *Lactobacillus* in the intestinal flora of diabetic mice was significantly reduced, the number of *Desulfovibrio* and *Enterococcus* was markedly increased compared to the normal people (Qin et al., 2012; Sato et al., 2014). *Firmicutes* and *Bacteroidetes* play an important role in the energy metabolism of host cells, and the proportion of *Firmicutes*to *Bacteroidetes* in many obese patients is much higher than that of the normal group (Hildebrandt et al., 2009). Our results are consistent with the conclusion, which demonstrated that the DM group had a greater relative abundance of *Firmicutes* and a lower relative abundance of *Bacteroidetes* compared to the CN group. PC and LS supplement significantly reduced the ratio of *Firmicutes* to *Bacteroides*, and HS did not change this proportion. Additionally, PC administration induced the occurrence of *Verrucomicrobia* at the phylum level (Figure 7a). Studies have shown that *Allobaculum* which belong to *Firmicutes* increased with the exacerbation of the pathological state of diabetes process and accompanied by high blood glucose levels in rats (Gu et al., 2016). In addition, we found that relative abundance of *Allobaculum* was higher in the HFD group than in the Hugan Qingzhi tablet (HQT) group, *Allobaculum* demonstrated significant (*p* < 0. 001) positive correlations with TG, TC, LDL‐C, IL‐6, IL‐1β, TNF‐α, and body weight and negative correlations with HDL‐C levels (Tang et al., 2018). Our results indicated that the mice with type II diabetes had a great relative abundance of *Allobaculum*. PC administration induced the occurrence of Akkermansia and decreased the relative abundance of *Allobaculum* at the genus level. Akkermansia can hinder the development of obesity, reduce fat accumulation, promote insulin sensitivity, and attenuate type II diabetes by affecting intestinal urea metabolism and energy absorption (Plovier et al., 2017). However, HS administration induced a particular increase in the relative abundance of *Mucispirillum*. LS and HS administration significantly increased the relative abundance of *odoribacter* and decreased the relative abundance of *Allobaculum* at the genus level (Figure 7b,c). Thus, SFN protect type II diabetes may involved in decreasing the relative abundance of *Allobaculum*.

## 4 CONCLUSION

Our results showed that SFN had a protective effect on type II diabetes. The supplement of SFN (2 m/kg, 10 mg/kg) increased serum insulin level, HOMA‐β index, and liver SOD and GSH activities as well as decreased the FBG, liver MDA, serum TC, TG, LDL‐C, and FGF21 levels. Remarkably, high dosage SFN (10 mg/kg) was able to decrease serum LPS level and increase the relative abundance of *Mucispirillum* at the genus level. In addition, SFN (2 m/kg, 10 mg/kg) was able to attenuate NAFLD, repair pancreas tissue, and decrease the relative abundance of *Allobaculum* at the genus level compared to diabetic mice. Oral supplement of SFN improves OGTT through easing NAFLD and repairing pancreas tissue in association with modulation of gut microbiota. The ease of NAFLD may be involved in enhancing liver antioxidant ability and decreasing FGF21 resistance by SFN. Our report provided a significant influence of SFN in the prevention and treatment of oxidative stress, NAFLD, and type II diabetes.

## References

[fsn32040-bib-0001] Axelsson, A. S. , Tubbs, E. , Mecham, B. , Chacko, S. , Nenonen, H. A. , Tang, Y. , Fahey, J. W. , Derry, J. M. J. , Wollheim, C. B. , Wierup, N. , Haymond, M. W. , Friend, S. H. , Mulder, H. , & Rosengren, A. H. (2017). Sulforaphane reduces hepatic glucose production and improves glucose control in patients with type 2 diabetes. Science Translational Medicine, 9(394), eaah4477 10.1126/scitranslmed.aah4477 28615356

[fsn32040-bib-0002] Beebe, C. (2003). Body weight issues in preventing and treating type 2 diabete. Diabetes Spectrum, 16(4), 261–266. 10.2337/diaspect.16.4.261

[fsn32040-bib-0003] Betteridge, D. J. (2000). What is oxidative stress? Metabolism‐Clinical and Experimental, 49(2), 3–8. 10.1016/S0026-0495(00)80077-3 10693912

[fsn32040-bib-0004] Calabrese, V. , Cornelius, C. , Dinkova‐Kostova, A. T. , Iavicoli, I. , Di Paola, R. , Koverech, A. , Cuzzocrea, S. , Rizzarelli, E. , & Calabrese, E. J. (2012). Cellular stress responses, hormetic phytochemicals and vitagenes in aging and longevity. BBA ‐ Molecular Basis of Disease, 1822(5), 753–783. 10.1016/j.bbadis.2011.11.002 22108204

[fsn32040-bib-0005] Cani, P. D. , Amar, J. , Iglesias, M. A. , Poggi, M. , Knauf, C. , Bastelica, D. , Neyrinck, A. M. , Fava, F. , Tuohy, K. M. , Chabo, C. , Waget, A. , Delmee, E. , Cousin, B. , Sulpice, T. , Chamontin, B. , Ferrieres, J. , Tanti, J.‐F. , Gibson, G. R. , Casteilla, L. , … Burcelin, R. (2007). Metabolic endotoxemia initiates obesity and insulin resistance. Diabetes, 56(7), 1761–1772. 10.2337/db06-1491 17456850

[fsn32040-bib-0006] Cani, P. D. , Neyrinck, A. M. , Fava, F. , Knauf, C. , Burcelin, R. G. , Tuohy, K. M. , Gibson, G. R. , & Delzenne, N. M. (2007). Selective increases of bifidobacteria in gut microflora improve high‐fat‐diet‐induced diabetes in mice through a mechanism associated with endotoxaemia. Diabetologia, 50, 2374–2383. 10.1007/s00125-007-0791-0 17823788

[fsn32040-bib-0007] Charlton, M. (2004). Nonalcoholic fatty liver disease: A review of current understanding and future impact. Clinical Gastroenterology and Hepatology, 2(12), 1048–1058. 10.1053/S1542-3565(04)00440-9 15625647

[fsn32040-bib-0008] Chen, P. , Zhang, Q. , Dang, H. , Liu, X. , Tian, F. , Zhao, J. , Chen, Y. , Zhang, H. , & Chen, W. (2014). Antidiabetic effect of *Lactobacillus casei* CCFM0412 on mice with type 2 diabetes induced by a high‐fat diet and streptozotocin. Nutrition, 30(9), 1061–1068. 10.1016/j.nut.2014.03.022 25102821

[fsn32040-bib-0009] Chen, X. C. , & Zhan, R. J. (2019). Protective effect of edgeworthia gardneri flos on pancreas in diabetes mellitus model rats induced by tacrolimus and its mechanism. Chinese Journal of Modern Applied Pharmacy, 36(16), 2008–2013. 10.13748/j.cnki.issn1007-7693.2019.16.005

[fsn32040-bib-0010] Choi, K.‐M. , Lee, Y.‐S. , Kim, W. , Kim, S. J. , Shin, K.‐O. , Yu, J.‐Y. , Lee, M. K. , Lee, Y.‐M. , Hong, J. T. , Yun, Y.‐P. , & Yoo, H.‐S. (2014). Sulforaphane attenuates obesity by inhibiting adipogenesis and activating the AMPK pathway in obese mice. Journal of Nutritional Biochemistry, 25, 201–207. 10.1016/j.jnutbio.2013.10.007 24445045

[fsn32040-bib-0011] Daems, C. , Welsch, S. , Boughaleb, H. , Vanderroost, J. , Robert, A. , Sokal, E. , & Lysy, P. A. (2019). Early treatment with empagliflozin and GABA improves beta‐cell mass and glucose tolerance in streptozotocin‐treated mice. Journal of Diabetes Research, 2019, Article ID: 2813489. 10.1155/2019/2813489 PMC6701376 31467926

[fsn32040-bib-0012] Eckburg, P. B. , Bik, E. M. , Bernstein, C. N. , Purdom, E. , Dethlefsen, L. , Sargent, M. , & Relman, D. A. (2005). Diversity of the human intestinal microbial flora. Science, 308, 1635–1638. 10.1126/science.1110591 15831718 PMC1395357

[fsn32040-bib-0013] Gao, Y. F. , Zhang, M. N. , Wu, T. C. , Xu, M. Y. , Cai, H. N. , & Zhang, Z. S. (2015). Effects of D‐pinitol on insulin resistance through the PI3K/Akt signaling pathway in type 2 diabetes mellitus rats. Journal of Agricultural and Food Chemistry, 63, 6019–6026. 10.1021/acs.jafc.5b01238 26063468

[fsn32040-bib-0014] Gu, C. Y. , Yang, Y. , Xiang, H. , Li, S. , Liang, L. , Sui, H. , & Lu, X. G. (2016). Deciphering bacterial community changes in zucker diabetic fatty rats based on 16S rRNA gene sequences analysis. Oncotarget, 7, 48941–48952. 10.18632/oncotarget.10597 27418144 PMC5226482

[fsn32040-bib-0015] Guerrero‐Beltran, C. E. , Calderon‐Oliver, M. , Pedraza‐Chaverri, J. P. , & Chirino, Y. I. (2012). Protective effect of sulforaphane against oxidative stress: Recent advances. Experimental and Toxicologic Pathology, 64, 503–508. 10.1016/j.etp.2010.11.005 21129940

[fsn32040-bib-0016] Haffner, S. M. , Greenberg, A. S. , Weston, W. M. , Chen, H. , Williams, K. , & Freed, M. I. (2002). Effect of rosiglitazone treatment on nontraditional markers of cardiovascular disease in patients with type 2 diabetes mellitus. Circulation, 106, 679–684. 10.1016/j.amjcard.2005.09.101 12163427

[fsn32040-bib-0017] Heiskanen, M. A. , Motiani, K. K. , Mari, A. , Saunavaara, V. , Eskelinen, J.‐J. , Virtanen, K. A. , Koivumäki, M. , Löyttyniemi, E. , Nuutila, P. , Kalliokoski, K. K. , & Hannukainen, J. C. (2018). Exercise training decreases pancreatic fat content and improves beta cell function regardless of baseline glucose tolerance: A randomised controlled trial. Diabetologia, 61(8), 1817–1828. 10.1007/s00125-018-4627-x 29717337 PMC6061150

[fsn32040-bib-0018] Hildebrandt, M. A. , Hoffmann, C. , Sherrill–Mix, S. A. , Keilbaugh, S. A. , Hamady, M. , Chen, Y. Y. , Knight, R. , Ahima, R. S. , Bushman, F. , & Wu, G. D. (2009). High‐fat diet determines the composition of the murine gut microbiome independently of obesity. Gastroenterology, 137, 1716–1724. 10.1053/j.gastro.2009.08.042 19706296 PMC2770164

[fsn32040-bib-0019] Kharitonenkov, A. , Shiyanova, T. L. , Koester, A. , Ford, A. M. , Micanovic, R. , Galbreath, E. J. , Sandusky, G. E. , Hammond, L. J. , Moyers, J. S. , Owens, R. A. , Gromada, J. , Brozinick, J. T. , Hawkins, E. D. , Wroblewski, V. J. , Li, D.‐S. , Mehrbod, F. , Jaskunas, S. R. , & Shanafelt, A. B. (2005). FGF‐21 as a novel metabolic regulator. Journal of Clinical Investigation, 115, 1627–1635. 10.1172/JCI23606 PMC1088017 15902306

[fsn32040-bib-0020] Lee, J. H. , Moon, M. H. , Jeong, J. K. , Park, Y. G. , Lee, Y. J. , Seol, J. W. , & Park, S. Y. (2012). Sulforaphane induced adipolysis via hormone sensitive lipase activation, regulated by AMPK signaling pathway. Biochemical and Biophysical Research Communications, 426, 492–497. 10.1016/j.bbrc.2012.08.107 22982310

[fsn32040-bib-0021] Li, S. , Yang, H. W. , & Chen, X. L. (2019). Protective effects of sulforaphane on diabetic retinopathy: Activation of the nrf2 pathway and inhibition of nlrp3 inflammasome formation. Experimental Animals, 68(2), 221–231. 10.1538/expanim.18-0146 30606939 PMC6511524

[fsn32040-bib-0022] Li, X. F. , Zhou, W. , Yin, B. X. , Fang, D. S. , Wang, G. , Zhao, J. X. , & Chen, W. (2018). Effects of mixed Lacteria Acid Bacteria which produced exopolysaccharide on Type 2 diabetic mice. Journal of Chinese Institute of Food Science and Technology, 018(009), 16–24. 10.16429/j.1009-7848.2018.09.003

[fsn32040-bib-0023] Liang, H. , & Yuan, Q. P. (2012). Natural sulforaphane as a functional chemopreventive agent: Including a review of isolation, purification and analysis methods. Critical Reviews in Biotechnology, 32, 218–234. 10.3109/07388551.2011.604838 21942647

[fsn32040-bib-0024] Liao, Z. Z. , Xu, L. Y. , Wang, J. N. , Tan, H. D. , & Wei, L. M. (2019). Protective effects of cinnamon polyphenol on streptozotocin‐induced diabetic mice. Journal of Xi’an Jiaotong University (Medical Science), 40(01), 170–174. 10.7652/jdyxb201901032

[fsn32040-bib-0025] Lim, E. L. , Hollingsworth, K. G. , Aribisala, B. S. , Chen, M. J. , Mathers, J. C. , & Taylor, R. (2011). Reversal of type 2 diabetes: Normalisation of beta cell function in association with decreased pancreas and liver triacylglycerol. Diabetologia, 54(10), 2506–2514. 10.3410/f.718381948.793494985 21656330 PMC3168743

[fsn32040-bib-0026] Ma, Q. , Li, Y. , Li, P. , Wang, M. , Wang, J. , Tang, Z. , Wang, T. , Luo, L. , Wang, C. , Wang, T. , & Zhao, B. (2019). Research progress in the relationship between type 2 diabetes mellitus and intestinal flora. Biomedicine & Pharmacotherapy, 117, 109–138. 10.1016/j.biopha.2019.109138 31247468

[fsn32040-bib-0027] Manaer, T. , Yu, L. , Zhang, Y. , Xiao, X. J. , & Nabi, X. H. (2015). Anti‐diabetic effects of shubat in type 2 diabetic rats induced by combination of high‐glucose‐fat diet and low‐dose streptozotocin. Journal of Ethnopharmacology, 169, 269–274. 10.1016/j.jep.2015.04.032 25922265

[fsn32040-bib-0028] Plovier, H. , Everard, A. , Druart, C. , Depommier, C. , Van Hul, M. , Geurts, L. , Chilloux, J. , Ottman, N. , Duparc, T. , Lichtenstein, L. , Myridakis, A. , Delzenne, N. M. , Klievink, J. , Bhattacharjee, A. , van der Ark, K. C. H. , Aalvink, S. , Martinez, L. O. , Dumas, M.‐E. , Maiter, D. , … Cani, P. D. (2017). A purified membrane protein from Akkermansia muciniphila or the pasteurized bacterium improves metabolism in obese and diabetic mice. Nature Medicine, 23(1), 107–113. 10.1038/nm.4236 27892954

[fsn32040-bib-0029] Qin, J. , Li, Y. , Cai, Z. , Li, S. , Zhu, J. , Zhang, F. , Liang, S. , Zhang, W. , Guan, Y. , Shen, D. , Peng, Y. , Zhang, D. , Jie, Z. , Wu, W. , Qin, Y. , Xue, W. , Li, J. , Han, L. , Lu, D. , … Wang, J. (2012). A metagenome‐wide association study of gut microbiota in type 2 diabetes. Nature, 490(7418), 55–60. 10.1038/nature11450 23023125

[fsn32040-bib-0030] Ryan, M. C. , Itsiopoulos, C. , Thodis, T. , Ward, G. , Trost, N. , Hofferberth, S. , O’Dea, K. , Desmond, P. V. , Johnson, N. A. , & Wilson, A. M. (2013). The Mediterranean diet improves hepatic steatosis and insulin sensitivity in individuals with non‐alcoholic fatty liver disease. Journal of Hepatology, 59(1), 138–143. 10.1016/j.jhep.2013.02.012 23485520

[fsn32040-bib-0031] Saeedi, P. , Petersohn, I. , Salpea, P. , Malanda, B. , Karuranga, S. , Unwin, N. , Williams, R. (2019). Global and regional diabetes prevalence estimates for 2019 and projections for 2030 and 2045: Results from the International Diabetes Federation Diabetes Atlas, 9th edition. Diabetes Research and Clinical Practice, 157, 107843 10.1016/j.diabres.2019.107843 31518657

[fsn32040-bib-0032] Sato, J. , Kanazawa, A. , Ikeda, F. , Yoshihara, T. , Goto, H. , Abe, H. , Komiya, K. , Kawaguchi, M. , Shimizu, T. , Ogihara, T. , Tamura, Y. , Sakurai, Y. , Yamamoto, R. , Mita, T. , Fujitani, Y. , Fukuda, H. , Nomoto, K. , Takahashi, T. , Asahara, T. , … Watada, H. (2014). Gut dysbiosis and detection of “live gut bacteria” in blood of Japanese patients with type 2 diabetes. Diabetes Care, 37(8), 2343–2350. 10.2337/dc13-2817 24824547

[fsn32040-bib-0033] Shaw, J. E. , Sicree, R. A. , & Zimmet, P. Z. (2010). Global estimates of the prevalence of diabetes for 2010 and 2030. Diabetes Research and Clinical Practice, 87(1), 4–14. 10.1016/j.diabres.2011.10.029 19896746

[fsn32040-bib-0034] Song, H. , Chu, Q. , Yan, F. Y. , Yang, Y. , Han, W. , & Zheng, X. D. (2015). Red pitaya betacyanins protects from diet‐induced obesity, liver steatosis and insulin resistance in association with modulation of gut microbiota in mice. Journal of Gastroenterology and Hepatology, 10.1111/jgh.13278 26699443

[fsn32040-bib-0035] Souza, C. G. , Motta, L. L. , Assis, A. M. , Rech, A. , Bruch, R. , Klamt, F. , & Souza, D. O. (2016). Sulforaphane ameliorates the insulin responsiveness and the lipid profile but does not alter the antioxidant response in diabetic rats. Food Function, 7(4), 2060–2065. 10.1039/c5fo01620g 27025193

[fsn32040-bib-0036] Tang, W. J. , Yao, X. R. , Xia, F. , Yang, M. T. , Chen, Z. J. , Zhou, B. J. , & Liu, Q. (2018). Modulation of the gut microbiota in rats by hugan qingzhi tablets during the treatment of high‐fat‐diet‐induced nonalcoholic fatty liver disease. Oxidative Medicine and Cellular Longevity, 4, 1–14. 10.1155/2018/7261619 PMC6323444 30671174

[fsn32040-bib-0037] Turnbaugh, P. J. , Ley, R. E. , Mahowald, M. A. , Magrini, V. , Mardis, E. R. , & Gordon, J. I. (2006). An obesity‐associated gut microbiome with increased capacity for energy harvest. Nature, 444(7122), 1027–1031. 10.1016/S0084-3741(08)70093-3 17183312

[fsn32040-bib-0038] Tushuizen, M. E. , Bunck, M. C. , Pouwels, P. J. , Bontemps, S. , Waesberghe, J. H. T. , Schindhelm, R. K. , & Diamant, M. (2007). Pancreatic fat content and β‐cell function in men with and without type 2 diabetes. Diabetes Care, 30(11), 2916–2921. 10.2337/dc07-0326 17666465

[fsn32040-bib-0039] Wang, Z. Z. , Ma, P. , Zuo, A. H. , Sun, T. L. , Peng, C. , Zhan, L. Q. , & Bao, W. Y. (2019). Hypoglycemic effect of apostichopus japonicus oligo‐petides in alloxan‐induced diabetic mice. Food Research and Development, 040(008), 85–90. 10.3969/j.issn.1005-6521.2019.08.015

[fsn32040-bib-0040] Xu, J. , & Gordon, J. I. (2003). Honor thy symbionts. Proceedings of the National Academy of Science of the United States of America, 100, 10452–10459. 10.1073/pnas.1734063100 PMC193582 12923294

[fsn32040-bib-0041] Yao, A. , Shen, Y. , Wang, A. , Chen, S. , Zhang, H. , Chen, F. , Chen, Z. , Wei, H. , Zou, Z. , Shan, Y. , & Zhang, X. (2015). Sulforaphane induces apoptosis in adipocytes via Akt/p70s6k1/Bad inhibition and ERK activation. Biochemical and Biophysical Research Communications, 465, 696–701. 10.1016/j.bbrc.2015.08.049 26296464

